# Thrombospondin-1, BIM and CFH polymorphisms and response to anti-VEGF treatment in neovascular age- related macular degeneration patients

**DOI:** 10.1371/journal.pone.0297135

**Published:** 2024-02-26

**Authors:** Christine M. Sorenson, Zafer Gurel, Yong-Seok Song, Kyle D. Peterson, Barbara A. Blodi, Nader Sheibani

**Affiliations:** 1 Department of Pediatrics, University of Wisconsin School of Medicine and Public Health, Madison, WI, United States of America; 2 McPherson Eye Research Institute, University of Wisconsin School of Medicine and Public Health, Madison, WI, United States of America; 3 Department of Human Oncology, University of Wisconsin School of Medicine and Public Health, Madison, WI, United States of America; 4 Department of Ophthalmology and Visual Sciences, University of Wisconsin School of Medicine and Public Health, Madison, WI, United States of America; 5 Department of Ophthalmology and Visual Sciences, Statistics Core, University of Wisconsin School of Medicine and Public Health, Madison, WI, United States of America; 6 Department of Cell and Regenerative Biology, University of Wisconsin School of Medicine and Public Health, Madison, WI, United States of America; 7 Department of Biomedical Engineering, University of Wisconsin School of Medicine and Public Health, Madison, WI, United States of America; City of Hope National Medical Center, UNITED STATES

## Abstract

Age-related macular degeneration (AMD) is a vision threatening disease in older adults. Anti-VEGF treatment is effective for the majority of neovascular AMD (nAMD) patients, although approximately 30% of nAMD patients have an incomplete response for unknown reasons. Here we assessed the contribution of single nucleotide polymorphisms (SNPs) in key angioinflammatory regulatory genes in nAMD patients with an incomplete response compared to those responsive to anti-VEGF treatment. A total of 25 responsive and 30 nAMD patients with an incomplete response to anti-vascular endothelial growth factor (anti-VEGF) treatment were examined for known SNPs that impact the structure and function of thromobospondin-1 (TSP1), Bcl-2-interacting mediator of cell death (BIM) and complement factor H (CFH). Plasma levels of C-C motif chemokine ligand 2 (CCL2/MCP1), TSP1 and VEGF were assessed by ELISA. Patients responsive to anti-VEGF treatment showed a significant increase in the TSP1 rs2228262 AA allele and a trend for the BIM (rs724710) CT allele. Consistent with previous reports, 42% of the patients responsive to anti-VEGF expressed the CC allele for CFH rs1061170. Although the CFH TT allele had similarly low prevalence in both groups, the TC allele tended to be more prevalent in patients with an incomplete response. Patients with an incomplete response also had increased plasma CCL2/MCP1 levels, consistent with the role increased inflammation has in the pathogenesis of nAMD. Our studies point to new tools to assess the potential responsiveness of nAMD patients to anti-VEGF treatment and suggest the potential use of anti-CCL2 for treatment of nAMD patients with an incomplete response to anti-VEGF.

## Introduction

Anti-vascular endothelial growth factor (VEGF) therapy is the standard of care in the treatment of neovascular age-related macular degeneration (nAMD) [1]. In a typical medical retina practice, 50–90% of all patients with nAMD receive intravitreal injections with anti-VEGF agents; these patients may receive up to 12 injections per year in the first 2–3 years and 4–6 injections per year after that. Unfortunately, up to 30% of patients can still have intraretinal fluid with vision loss after 1 year of therapy [2–4]. Even though anti-VEGF treatment is effective for many nAMD patients, its use comes with an increased treatment burden and risk of vision complications including retinal atrophy and endophthalmitis. The inability to identify patients who will have an incomplete response to anti-VEGF treatment has a far-reaching impact, affecting patients, their families, and the healthcare system.

Although VEGF expression is essential for maintenance of the retinal pigment epithelium (RPE), its increased expression contributes to neovascularization and leakiness of blood vessels. RPE atrophy is noted in about 20–25% patients receiving anti-VEGF treatment, further demonstrating the essential role VEGF plays in tissue development and homeostasis [5, 6]. Optimal treatment with anti-VEGF therapy for ocular neovascularization requires balancing VEGF inhibition to reduce vessel permeability and pathological neovascularization, without causing RPE atrophy. This indicates that the efficacy of anti-VEGF treatment may vary based on genetic polymorphisms in key angioinflammatory regulatory factors. Therefore, development of biomarkers to assess a patient’s responsiveness to anti-VEGF prior to onset of treatment would be beneficial.

Aberrant regulation of angiogenesis and inflammatory processes plays a central role during the pathogenesis of many ocular diseases including nAMD. The visual deficit that initially arises from retinal degeneration (dry AMD) is often complicated by the secondary effects of choroidal neovascularization (CNV). This is principally driven by VEGF, causing progression to wet or nAMD [7]. Ocular vascular homeostasis is maintained by a balanced production of positive and negative angioregulatory factors. During development, this balanced angioregulatory factor production facilitates normal vascularization and maintenance. However, disruption of this balance by inflammation, oxidative stress, or ischemia can lead to pathological neovascularization and/or vascular rarefaction. Thus, understanding how dysregulation of angioinflammatory regulatory factors promotes nAMD will aid in its treatment.

Thrombospondins are a family of matricellular proteins that play essential roles during remodeling and re-organization of extracellular matrix components by influencing cell proliferation, migration, and apoptosis [8]. Thrombospondin-1 (TSP1) is a potent endogenous inhibitor of angiogenesis and modulator of immunity [9]. Its expression in the endothelium promotes a quiescent differentiated phenotype [10, 11]. Decreased TSP1 expression exacerbates ocular neovascularization [12]. However, the contribution of genetic variations affecting the structure and function of TSP1 during the pathogenesis of ocular disease in humans has not been extensively studied. TSP1 polymorphisms are associated with reduced TSP1 plasma levels, altered calcium binding capacity and stability, and increased risk of myocardial infarction [13–15]. TSP1 single nucleotide polymorphism (SNP; rs1478604) is linked to decreased TSP1 expression with a subsequent increase in interleukin 1β (IL1β) expression in chronic ocular surface inflammation following refractive surgery [16] and an increased risk of corneal allograft rejection [17]. Since TSP1 expression also affects inflammatory processes, TSP1 polymorphisms should facilitate sustained inflammation in nAMD leading to an incomplete response to anti-VEGF treatment. This notion is further supported by result of a recent study that showed complement factor H (CFH) polymorphisms, a risk factor for nAMD, attenuates TSP1 interaction with its receptor (CD47) on mononuclear phagocytes delaying their clearance [18, 19].

Interaction of TSP1 with CD47 and CD36 on the endothelium mediates the antiangiogenic activity of TSP1 through modulation of NO-cGMP mediated angiogenic activities of endothelial cells from various vascular beds [20, 21]. Our studies of ocular vasculature, both retinal and choroidal vasculature, have revealed significant differences in the properties of their endothelial cells. They specifically contrast in their cell autonomous responses to TSP1 expression and its impact on their anti-inflammatory properties [10, 22]. This was further emphasized in a recent review that discussed the impact of aging on TSP1 expression and activity in vascular bed of many tissues [23], which is in contrast to reports in the choroid with aging and pathogenesis of AMD [24].

The ability of macrophages to undergo apoptosis is essential for immune cell clearance. The anti-inflammatory activity of TSP1 in macrophages is linked to its ability to induce clearance of macrophages through interaction with CD47 [18, 19]. Furthermore, Bcl-2-interacting mediator of cell death (BIM) is a Bcl-2 homology 3 domain (BH3)-only Bcl-2 family member that promotes apoptosis when presented with a death stimulus. While BIM plays a role in immune cell clearance by eliminating unnecessary cells, its function in the choriocapillaris remains unclear. Our recent preclinical studies showed that mice lacking Bim expression are unable to effectively clear mononuclear phagocytes leading to increased subretinal fibrosis and scarring during CNV [25]. BIM polymorphisms that decrease BIM expression are well known to cause patient resistance to many conventional cancer treatments including anti-VEGF [26–28]. Thus, determining the role TSP1, BIM, and CFH polymorphisms play in nAMD pathophysiology and treatment responsiveness should decrease treatment burden for nAMD patients.

Here we assessed the association of TSP1, BIM, and/or CFH SNPs with nAMD patients’ responsiveness to anti-VEGF therapy. A significant increase in the frequency of TSP1 SNP rs2228262 AA allele in patients that were responsive to therapy compared to those with an incomplete response was observed. Also noted was a trend for the BIM (rs724710) CT allele to be associated with responsiveness to treatment. Given the role inflammation plays in the pathogenesis of nAMD, plasma levels of C-C motif chemokine ligand 2 (CCL2/MCP1) and VEGF were assessed. A significant increase in CCL2/MCP1 plasma levels in patients with an incomplete response to treatment was noted. These patients also had a trend of increased VEGF plasma levels. Thus, the studies presented here begin to address the potential interplay between TSP1 and BIM SNPs influencing responsiveness to anti-VEGF for nAMD treatment and implicate an important role for the anti-inflammatory activity of TSP1.

## Materials and methods

### Ethics statement and patient samples

This study was approved by the University of Wisconsin Institutional Review Board (approvals: 2019–0096, 2019-0096-CP001, 2019-0096-CP002, 2019-0096-003), with written informed consent obtained from all participants. Participants were identified and recruited during a regular clinic visit from 11/5/2019 through12/23/2020 with Dr Blodi. The Clinical Eye Research Unit (CERU) in the Department of Ophthalmology and Visual Sciences obtained informed consent during a routine clinical visit from all identified and interested participants prior to research procedures (collecting blood samples and data). The CERU coded all collected data and the to the code was stored securely within the CERU and not with the laboratory preforming analysis. All information was collected and secured in compliance with the Health Insurance Portability and Accountability Act and the study conducted according to the tenets set by the Declaration of Helsinki.

Blood samples were obtained from nAMD patients seen in the Department of Ophthalmology and Visual Sciences Clinic at the University of Wisconsin School of Medicine and Public Health in which informed consent for the study was obtained. All samples (n = 55) were collected in a 3 ml tube and were obtained before anti-VEGF injection at a routine clinic visit. Samples were sequenced for BIM rs724710, TSP1 rs1478604, rs2228262 and CFH rs1061170 SNPs as delineated in Table 1. Given the prevalence of the BIM polymorphism is ~30%, a sample size of at least 20 patients in each group was estimated to provide 80% power to detect and effect size of 0.16 at a level of significance of 0.05, when using a chi-square test of equal proportions in three groups. Calculations were performed using nQuery Advisor. Please note that this study took place during the COVID pandemic, which restricted patient access and participation.

**Table 1:** SNP for TSP1, BIM and CFH analyzed. The location and allelic change are noted.

Gene	Reference SNP	Location	Allele
TSP1	rs1478604	5’UTR	T→C
TSP1	rs2228262	Type III repeat	A→G
BIM	rs724710	BH3 domain	C→T
CFG	rs1061170	Heparin domain	T→C

A retina specialist divided patients into two groups, those responsive versus those with an incomplete response to anti-VEGF treatment. Patients responsive to anti-VEGF treatment were defined due to the absence of intraretinal and subretinal fluid on optical coherence tomography after 1 year of treatment. The patients with an incomplete response were defined as having persistence of fluid following 1 year of anti-VEGF treatment. Artificial intelligence (AI) technologies were not utilized in these studies or manuscript preparation.

### DNA preparation

Patient samples were collected in a masked manner. Blood samples (3 ml) were collected in BD vacutainer tube (Becton, Dickinson and Company, Franklin Lakes, NJ) and frozen at -80°C until DNA extracted. DNA was extracted using the Gentra Puregene Blood Core kit A purification kit (#158467; Qiagen, Hilden, Germany) as directed by the supplier. The area of interest was amplified using Platinum SuperFi DNA polymerase (#12368002; Invitrogen ThermoFisher, Carlsbad, CA) with BIM or TSP1 primers in Table 2 and sent for Sanger sequencing at the UW Biotechnology Center using BigDye reaction. The CFH rs1061170 SNP was assessed using TaqMan^TM^ CFH SNP Genotyping Assay (ThermoFisher; #7119815–1) and analyzed by qPCR (50°C for 2 min, 95°C for 2 min, then 95°C for 10 sec, 60°C for 30 sec and repeated 40 times).

**Table 2:** PCR and sequencing primers used for BIM and TSP1 SNPs.

Gene	PCR Primer	PCR Steps	PCR Product Size	Sequencing Primer
BIM rs724710	F:TGGTGTTTGCAGACTTGAGC R:GAAGGGGAGGGTGTGAGC	98° 30s, [98° 10s, 64° 10s, 72° 30s]x34, 72° 5m, 10° Pause	800 bp	AGTTATGTAGAAGACTCTGCCACTCT
TSP1 rs1478604	F:CAGGCATTCCGGGAGATCAG R:GAGAGTGTAGGTTCCGGGGT	98° 30s, [98° 10s, 66° 10s, 72° 20s]x34, 72° 5m, 10° Pause	640 bp	GTTCTAGTGCTCCCAAGCCC
TSP1 rs2228262	F:ACGCCAAGTGCAACTACCTG R:TTAGTGCCCCTCTCCCTTTGG	98° 30s, [98° 10s, 61° 10s, 72° 20s]x34, 72° 5m, 10° Pause	303 bp	GCAACTACCTGGGCCACTAT

### ELISA

Patient plasma samples were aliquoted and frozen at -80°C until the specific ELISA was performed. For the ELISA, plasma was appropriately diluted with calibrator diluent and levels for TSP1, VEGF, or MCP-1 were assessed as indicated by the vendor utilizing Human Thrombospondin-1 Quantikine ELISA kit (DTSP10; R&D Systems, Minneapolis, MN), Human VEGF Quantikine ELISA kit (DVE00; R&D Systems) or Human CCL2/MCP1 Quantikine ELISA kit (DCP00; R&D Systems).

### RNA purification and real time qPCR analysis

Wild type and TSP1 -/- Immortomice were generated, the choroid harvested, rinsed with serum-free Dulbecco’s Modified Eagles’s Medium (DMEM) and endothelial cells isolated as we previously described [22]. Choroidal endothelial cells were maintained in a tissue culture incubator at 33°C with 5% CO_2_ in DMEM containing 10% fetal bovine serum (FBS), 2 mM L-glutamine, 2 mM sodium pyruvate, 20 mM HEPES, 1% nonessential amino acids, 100 μg/ml streptomycin, 100 U/ml penicillin, 55 U/ml heparin (Sigma, St. Louis, MO) and 44 U/ml interferon-γ (INF-γ; R&D, Minneapolis, MN) on 60 mm tissue culture dishes coated with 1% gelatin (Sigma #G1890). The cells were grown to approximately 70% confluence for use in the experiments. Total RNA from murine choroidal endothelial cells, wild type and TSP1 -/-, was extracted using mirVana PARIS kit (Invitrogen). Sprint RT Complete-Double PrePrimed kit (Clontech, Mountain View, CA) was used for cDNA synthesis with total RNA (1 μg) extracted from cells using a mirVana PARIS kit (Invitrogen). For a template, cDNA (1 μl each diluted 1∶10) was used in qPCR assays which were performed in triplicate of three biological replicates on a Mastercycler Realplex (Eppendorf) with a SYBR-Green qPCR Premix (Clontech). Amplification parameters were as follows: 95°C for 2 min; 40 cycles of amplification (95°C for 15 sec, 60°C for 40 sec); dissociation curve step (95°C for 15 sec, 60°C for 15 sec, 95°C for 15 sec). Primer sequences used; MCP-1 5’-GTCTGTGCTGACCC CAAGAAG-3’ (forward) and MCP-1 5’-TGGTTCCGATCCAGGTTTTTA-3’ (reverse), IL6 5’- CAACCAC GGCC TTCCCTACT-3’ (forward) and IL-6 5’-TTGGGAGTGGTATC CTCTGTGA-3’ (reverse), IL1β 5’- AGCTTCAGGCAGGCAGTATC-3’ (forward) and 5’-TGTCCTCATCC TGGAA GGTC-3’ (reverse), and RpL13a were 5′-TCTCAAGGTTGT TCGGCTGAA-3′ (forward) and Rpl13a 5′-CCAGACGCCCCAGGTA-3′ (reverse).

Standard curves from known quantities of each target gene with linearized plasmid DNA were generated. We used ten times dilution series for each known target, which we amplified using SYBR-Green qPCR. The linear regression line for DNA (ng) was then determined from relative fluorescent units (RFU) at a threshold fluorescence value (Ct). We were able to quantify gene targets from cell extracts by comparing the RFU at the Ct to the standard curve, normalized by the simultaneous amplification of RpL13a, a housekeeping gene.

### Statistical and data analysis

To analyze ELISA data and the qPCR data we evaluated statistical differences using the Student’s unpaired *t*-test (2-tailed). Mean ± standard error is shown. P< 0.05 was considered significant. Genetic association models were used to assess the influence of the studied SNPs on nAMD patients’ responsiveness to anti-VEGF treatment. Allele variations include homozygous wildtype (MM), heterozygous mutations (Mm), and homozygous mutations (mm), where the genetic contrasts considered were dominant (MM vs Mm + mm), recessive (MM + Mm vs mm), and co-dominant (MM > Mm > mm) [29]. Taking the moderate sample size into account, Bayesian logistic regressions were performed to obtain odds ratios as the parameters of interest and 95% highest density credible intervals (CI) to represent uncertainty. Posterior probability of direction (PD) was used to estimate the probability that the odds ratios were strictly above or below 1 respective to the direction of the posterior mean. Patient sex and age at time of visit were considered as covariates in the regression models but were removed due to sub-optimal model fit.

Weakly informative normal priors were specified for each covariate. A Markov chain Monte Carlo Gibbs sampler with three chains was used to draw 50,000 iterations from the joint posterior distribution after 25,000 burn-in iterations and thinning by 25 iterations. Convergence and mixing were assessed for each model via trace plots for visual confirmation as well as the Gelman-Rubin diagnostics for quantitative justification. We then performed a sensitivity analysis to assess the reliability of posterior inferences (Table 3).

**Table 3:** Sensitivity analysis of the Bayesian logistic regression genetic association models. Prior sensitivity > 0.05 indicates informative prior (*). Likelihood sensitivity < 0.05 indicates noninformative likelihood (^†^).

Gene/SNP	Genetic Association Model	Comparison Group	Prior Sensitivity	Likelihood Sensitivity
TSP1 rs1478604 (T/C)	Dominant	T vs C+CT	0.01	0.08
	Recessive	CT+T vs C	0.01	0.10
	Co-dominant	T vs CT vs C	0.02	0.08
TSP1 rs2228262 (A/G)	Dominant	A vs G+AG	0.02	0.09
	Recessive	AG+A vs G	0.14*	0.03^†^
	Co-dominant	A vs AG vs G	0.07*	0.03^†^
BIM rs724710 (C/T)	Dominant	C vs T+CT	0.01	0.11
	Recessive	CT+C vs T	< 0.01	0.10
	Co-dominant	C vs CT vs T	0.01	0.09
CFH rs1061170 (T/C)	Dominant	T vs C+CT	< 0.01	0.09
	Recessive	CT+T vs C	< 0.01	0.08
	Co-dominant	T vs CT vs C	0.01	0.11

Investigating the sensitivity of the posterior distribution to perturbations of both the prior and likelihood is critical to the Bayesian framework [30]. We evaluated model sensitivity by using the cumulative Jensen-Shannon distance as a metric to compare the divergence between the base and perturbed prior (and likelihood) distributions [31]. This robustness check ensures our findings were resilient to underlying changes in both prior beliefs and observed data likelihoods, and that the conclusions drawn from our Bayesian framework remain reliable for interpretations considering the moderate sample size.

Most of the genetic association models show evidence for reliable inference. However, the TSP1 rs2228262 recessive and co-dominant models demonstrate prior sensitivity with low likelihood sensitivity, which indicates the likelihood is weakly informative in relation to the prior. Given that the assigned weakly informative priors dominate the posterior, suggests there may not be enough observations to make reliable comparisons for these two models.

## Results

### Thrombospondin-1 polymorphisms

TSP1 is known for its ability to influence angiogenesis and inflammation. Genetic variation in the 5’-UTR of TSP1 is associated with its decreased expression [16]. Decreased TSP1 expression is accompanied by increased inflammation and IL1β expression [16]. Here we sequenced TSP1 SNPs (rs1478604 (5’-UTR) and rs2228262 (Type III repeats) in patients that were responsive and those with an incomplete response to anti-VEGF treatment. The age of onset ranged from 58–89 years of age while the age at visit ranged from 60–94 years of age. In the responsive group there were 14 females and 11 males, while in the group with an incomplete response there were 22 females and 8 males (Table 4).

**Table 4:** Age of onset and visit for male and female patients either responsive to anti-VEGF treatment or those with an incomplete response.

	Responsive	Incomplete Response
**Age Onset (years)**	58–89	59–89
**Age Visit (years)**	65–94	60–93
**Number female/male**	14/11	22/8
**<75 at Onset female/male**	7/6	14/2
**>75 at Onset female/male**	6/4	8/5

**Fig 1:**
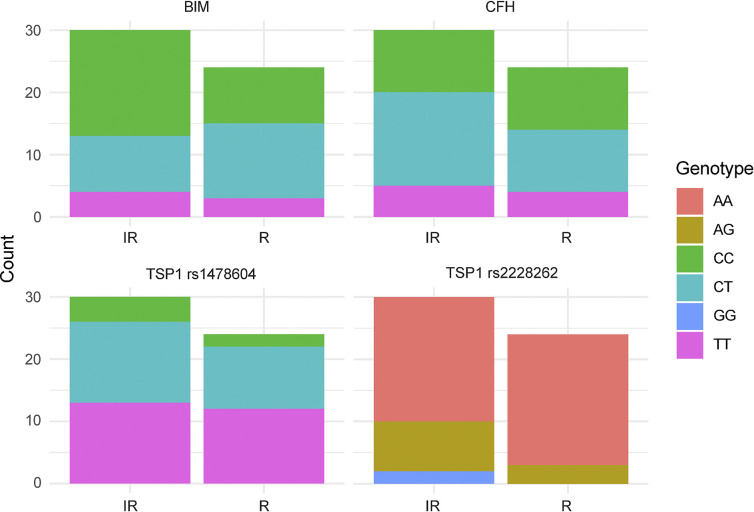
TSP1, BIM, and CFH prominence in nAMD patients both responsive and those with an incomplete response to anti-VEGF therapy. DNA was sequenced from nAMD patients responsive and those with an incomplete response to anti-VEGF therapy for TSP1 rs1478604 and rs2228262, BIM rs724710 and CFH rs1061170. The allele genotypes for responsive and those with an incomplete response are noted in color in the key on the left. Please note that patients with TSP1 rs2228262 AA allele were significantly increased in patients responsive to treatment.

The TSP1, BIM, and CFH SNPs examined, location and allelic changes are delineated in Table 1. Patients responsive to treatment had a significantly higher AA allele frequency in the TSP1 SNP rs2228262 (Fig 1, Table 5). The odds of responsiveness were 5-fold greater on average for the homozygous wildtype (A) carriers compared to the heterozygous and homozygous mutation carriers (Table 6), with an estimated 68% (95% CI: 37%– 89%) to respond compared to 31% (95% CI: 8%– 63%), respectively. Of the patients screened, none of the patients responding to treatment had the GG allele in TSP1 SNP rs2228262. The number of patients with TT and TC alleles in TSP1 SNP rs1478604 was not significantly different between responsive patients and those with an incomplete response (Fig 1, Table 5). Thus, patients responding to anti-VEGF treatment had a higher AA allele frequency in the TSP1 SNP rs2228262 and did not have the GG allele.

**Table 5:** Allelic changes noted in TSP1, BIM and CFH SNPs in responsive and those patients with an incomplete response to anti-VEGF therapy.

SNP	Gene	Allele	Responsive		Incomplete Response	
			Number	%	Number	%
rs1478604	TSP1	T	13	52	13	43
		C	2	8	4	14
		T/C	10	40	13	43
rs2228262	TSP1	A	22	88	20	67
		G	0	0	2	7
		A/G	3	12	8	26
rs724710	BIM	C	9	36	17	57
		T	3	17	4	13
		C/T	13	53	9	30
rs1061170	CFH	T	4	18	5	17
		C	10	42	10	33
		T/C	10	42	15	50

**Table 6:** Odds of response to anti-VEGF treatment according to genetic association models. OR = odds ratio, CI = credible interval, PD = probability of direction. Co-dominant odds ratio corresponds to homozygous wildtype versus homozygous mutations.

Gene/SNP	Genetic Association Model	Comparison Group	OR	95% CI	PD
TSP1 rs1478604 (T/C)	Dominant	T vs C+CT	0.63	[0.15, 2.37]	0.76
	Recessive	CT+T vs C	0.87	[0.10, 8.52]	0.55
	Co-dominant	T vs CT vs C	0.44	[0.04, 5.81]	0.75
TSP1 rs2228262 (A/G)	Dominant	A vs G+AG	5.00	[1.03, 32.00]	0.97
	Recessive	AG+A vs G	0.03	[0.0, 3.25]	0.93
	Co-dominant	A vs AG vs G	39.25	[0.76, 2227.81]	0.97
BIM rs724710 (C/T)	Dominant	C vs T+CT	0.41	[0.12, 1.30]	0.93
	Recessive	CT+C vs T	0.90	[0.17, 5.02]	0.54
	Co-dominant	C vs CT vs T	0.61	[0.10, 3.89]	0.70
CFH rs1061170 (T/C)	Dominant	T vs C+CT	0.96	[0.19, 4.89]	0.51
	Recessive	CT+T vs C	1.54	[0.48, 4.79]	0.78
	Co-dominant	T vs CT vs C	0.50	[0.08, 3.43]	0.77

### BIM polymorphisms

BIM is a pro-apoptotic Bcl-2 family member that acts in opposition to Bcl-2 in the vasculature [32]. BIM expression is necessary for many therapeutic regimens that remove unwanted cells via apoptosis. Individuals carrying a C→T substitution (c456C>T; rs724710) within the BIM BH3 domain (death initiating domain) are reported to have lower basal BIM expression [33]. We examined BIM rs724710 SNP in nAMD patients responsive to treatment and those with an incomplete response. Patients with an incomplete response tended to have a CC allele, although it did not reach significance with our sample size (Fig 1, Table 5). We also noted that a greater portion of responsive patients had Bim CT allele as well as a corresponding TSP1 SNP rs1478604 TT allele (9 of 12 patients with Bim CT allele) compared to patients with an incomplete response (3 of 9 patients with Bim CT allele). All of these patients had TSP1 SNP rs2228262 AA alleles. BIM rs724710 carriers showed a lack of response on average; being homozygous wildtype (CC) rendered a lower odds of response to anti-VEGF therapy (OR = 0.41, 95% CI = .012–1.30, PD = 0.93) compared to the heterozygous and homozygous mutations group (Table 5), with an estimated 15% (95% CI: 3%– 41%) to respond compared to 31% (95% CI: 8%– 63%), respectively.

### CFH polymorphisms

CFH has been identified as a risk allele for AMD, with polymorphisms (Y402H; rs1061170) in this gene linked to persistent inflammation [34]. To address the possibility that CFH polymorphisms together with BIM and/or TSP1 polymorphisms enhance inflammation by further reducing inflammatory cell clearance and treatment efficacy, we genotyped patient samples for the CFH SNP rs1061170. CFH rs1061170 homozygous CC allele has been associated with a higher risk of advanced AMD [35–37] and is reported to be associated with a good response to anti-VEGF treatment in 41.3% of patients [38]. However, our results lack sufficient evidence to confirm these results. Of anti-VEGF responsive patients, 42% had the CC allele compared to 33% of patients with an incomplete response (Fig 1, Table 5). The TT allele had similarly low prevalence in both groups, with the TC allele tending to be more prevalent in the patient population with an incomplete response to anti-VEGF therapy. Thus, our study confirms that ~42% of the patient population responsive to anti-VEGF treatment express the CC allele for CFH rs1061170.

### Increased MCP-1 plasma levels in patients with incomplete response to anti-VEGF

**Fig 2:**
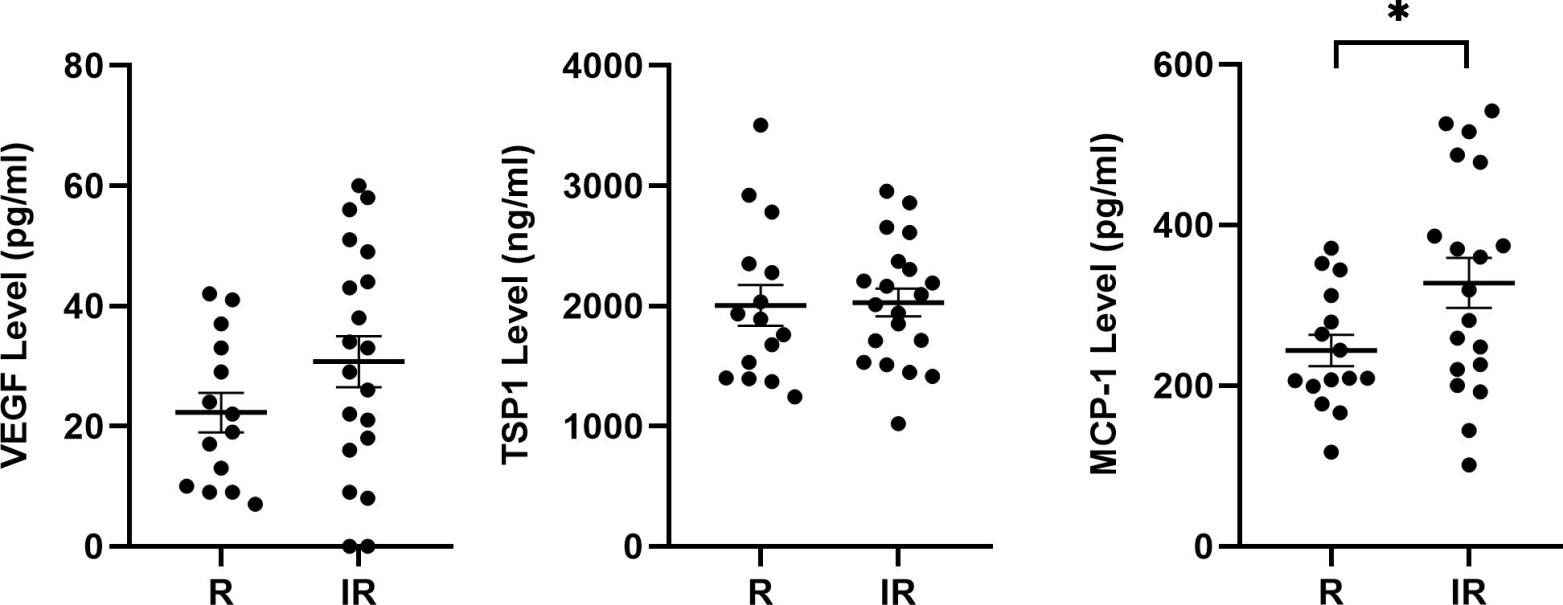
Increased CCL2/MCP-1 levels in patients with an incomplete response. Patient plasma levels of CCL2/MCP-1, VEGF and TSP1 were assessed by ELISA. Levels were assessed in patients responsive (R) and those with an incomplete response (IR) to anti-VEGF therapy. Please note that patients with an incomplete response to therapy had significantly increased CCL2/MCP-1 levels. (*P<0.05; n≤15; Student’s unpaired *t*-test (2-tailed) was utilized).

Inflammation plays a central role in the pathogenesis of AMD. TSP1 and BIM play key roles in immunosuppression and macrophage clearance in CNV [18, 25]. TSP1 and/or BIM polymorphisms, which decrease their protein levels, should increase macrophage numbers and production of inflammatory mediators. Since VEGF, a known culprit driving the pathogenesis of nAMD, is a mediator of inflammation, we assessed the impact anti-VEGF therapy had on plasma levels of key pro-inflammatory mediators including VEGF, CCL2/MCP-1, and TSP1 in anti-VEGF responsive and those patients with an incomplete response. Patients that had an incomplete response to anti-VEGF treatment demonstrated significantly increased CCL2/MCP-1 plasma levels (Fig 2). In patients with an incomplete response, VEGF levels had a trend to be increased although it did not reach significance with our sample size (Fig 2). TSP1 plasma levels were similar in both groups.

### Increased inflammatory mediator expression in choroidal endothelial cells lacking TSP1 expression

**Fig 3:**
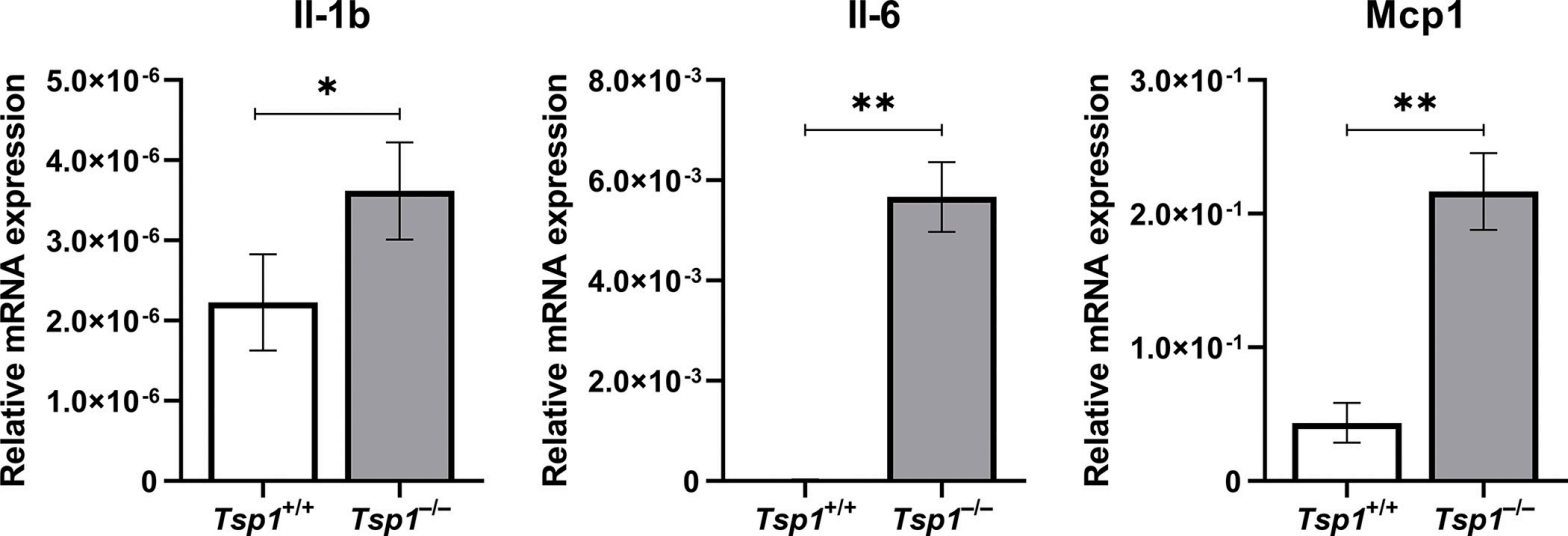
Increased inflammatory mediators TSP1^-/-^ choroidal endothelial cells. The expression levels of various inflammatory mediators were assessed by qPCR analysis for MCP-1, IL-6 and Il-1b. These experiments were repeated three times with two different isolations of murine choroidal endothelial cells with similar results. Please note a significant increase in the levels of MCP-1 and IL6 (*P<0.05; **P <0.01; n = 6; Student’s unpaired *t*-test (2-tailed) was utilized). RpL13A, 60S ribosomal protein L13a.

To assess the interrelationship between TSP1 and inflammatory mediators we determined the expression of CCL2/MCP-1, IL6, and IL1β in choroidal endothelial cells isolated from wild-type and mice lacking TSP1 (TSP1^-/-^). Fig 3 shows that choroidal endothelial cells lacking TSP1 expression had increased expression of CCL2/MCP-1, IL6, and IL1β. Thus, lack of TSP1 expression increased inflammatory mediator expression in choroidal endothelial cells.

## Discussion

Patient responsiveness to drug treatment is essential to halt disease progression. In many disease states, the standard available treatment does not have an optimal outcome for all patients. Typically, the issue is multifactorial leaving it difficult to predict pathogenesis and treatment effectiveness. However, some progress has been made, particularly in assessing sub-optimal outcomes for cancer treatment. Responsiveness to anti-VEGF, corticosteroids, and tyrosine kinase inhibitors hinge on BIM function, with BIM polymorphisms negating effective treatment [27, 33, 39]. The synonymous BIM polymorphism (rs724710) has a CC allele frequency that varies from 43–87% depending on the population background [28, 33, 40], with higher basal BIM expression reported in individuals having this CC allele [27, 33]. The importance of gauging treatment dose with allelic expression is typified by patients with a CC allele having a greater sensitivity to corticosteroid treatment and an increased incidence of osteonecrosis [39]. RPE atrophy is noted in about 20–25% of AMD patients receiving anti-VEGF treatment [5, 6]. Given the BIM CC allele increases basal BIM expression, perhaps a suboptimal response to anti-VEGF treatment and/or RPE atrophy is associated with the CC allele in BIM. Consistent with this supposition, we show a trend to an incomplete response in patients with the CC allele compared to responsive patients the trend to the CT allele. These studies further support the premise that dosage for optimal response to therapies, which rely on BIM expression, may be dependent on the BIM SNP expressed (CC vs CT allele).

BIM and VEGF act in a reciprocal manner. VEGF expression down-regulates BIM expression contributing to the formation of new leaky vessels during CNV. However, it is not known whether BIM apoptotic or non-apoptotic properties are contributing factors. The impact BIM has both on apoptosis and the extracellular matrix milieu is integral to its role during vascular remodeling, regression and resolution of neovascularization [32, 41]. Anti-VEGF therapy should increase BIM expression to a level that will squelch pathologic neovascularization and vessel leakiness without compromising the RPE or choroidal and retinal vascular integrity. Thus, balanced BIM and VEGF expression is essential to maintain vision. Too little or too much of either, as occurs as the result of polymorphisms, can lead to pathological neovascularization or vessel rarefaction. BIM expression in this context is important because the aim is to induce apoptosis of the targeted cells.

Much effort has focused in recent years on identifying genes that are associated with susceptibility to AMD. CFH rs1061170 (Y402H) is a risk allele identified to be associated with a higher risk of advanced disease in patients with the homozygous CC allele [35–37]. Several studies have also assessed the efficacy of anti-VEGF in patients with various CFH alleles. One study demonstrated nearly 42% of patients responding to anti-VEGF therapy had the CC allele [38], which is in line with the results obtained here. The study by Chen and colleagues concluded that anti-VEGF treatment was less effective in patients with the CC allele [38], while a study in a Brazilian cohort saw less visual improvement in the CC allele group [42]. Here, a trend for patients with an incomplete response to anti-VEGF treatment was seen to be heterozygous at this allele. In contrast, responsive patients had a similar likelihood of having the CC or TC allele, with about 20% of the responsive group having a TT allele. This variation may be due to the patient population background in Wisconsin, although in the future, increased patient enrollment numbers would aid in determining the significance of these trends.

TSP1 is a matricellular protein that is expressed in multiple ocular cell types. TSP1 is well known for its ability to inhibit inflammation and angiogenesis. In the eye it regulates ocular surface inflammation (dry eye disease) [43]. Its expression is downregulated in ocular disease with a neovascular component including diabetic retinopathy and uveal melanoma [44, 45]. We previously showed that vitreous and aqueous humor from eyes of various species including human, mouse, rat, and bovine contain TSP1 [44]. Our studies using mice deficient in TSP1 demonstrated that TSP1 expression is essential for ocular vascular homeostasis and its absence results in increased retinal vascular density during development and enhanced diabetic retinopathy [46, 47]. In the patient population we observed significantly more patients responsive to anti-VEGF therapy had the TSP1 AA allele SNP rs2228262 with none of the responsive patients having the GG allele of this SNP. In SNP rs2228262, changing A to G results in an amino acid change (asparagine to serine) in the calcium binding domain significantly impacts TSP1 structure and function. This substitution is noted in about 8–10% of the European population, similar to our responsive anti-VEGF treatment group (12%). However, the patients with an incomplete response to treatment 33% had a homo- or heterozygous substitution. Substitution of serine lowers calcium binding capacity decreasing TSP1 conformational stability but enhancing platelet aggregation [15, 48, 49]. Thus, the rs2228262 polymorphism is thought to correlate with vascular dysfunction [50]. This is consistent with our results here showing decreased response to anti-VEGF in nAMD patients with this polymorphism.

Conjunctival (CIC) specimens from patients with the TSP1 minor CC allele SNP rs1478604 are reported to have lower TSP1 expression than the major allele SNP (TT) [16]. Here we did not see a significant difference in plasma TSP1 levels between the responsive group and those with an incomplete response. Only had one patient in the responsive and 3 in the incomplete response group that had the minor CC allele SNP rs1478604. A previous study showed decreased TSP1 expression in peripheral blood samples from control patients carrying the CC allele. In this group about ~7% of the patients had the CC allele (3 patients) [51]. Also ~7% of our responsive group had the CC allele (1 patient) plus an additional 3 patients with an incomplete response (~16%). Although the plasma levels from all patients with the rs1478604 CC allele in our study (4 patients both responsive and incomplete response) had a mean plasma TSP1 level lower than their other allelic counterparts, it did not reach significance. This could be attributed to the low numbers of patients with the CC allele. It also remains unknown as to whether local TSP1 levels are significantly different than plasma levels in these patients. Here we noted choroidal endothelial cells lacking TSP1 expression had increased levels of inflammatory cytokines. Our previous studies with choroidal endothelial cells noted similar levels of VEGF expression in wild-type and TSP1 -/- cells [22]. We have also shown that although retinal endothelial cells from TSP1-/- mice are more migratory and proliferative [10], choroidal endothelial cells were less proliferative and migratory but exhibited a more inflammatory phenotype with significant upregulation on iNOS expression [22]. Therefore, the mechanisms of TSP1 action in choroidal endothelial cells through its receptors, namely CD36 and CD47, might be significantly different than endothelial cells from vascular beds of other tissues. In fact, we have noted significantly higher levels of CD36 expression in choroidal endothelial cells compared to retinal endothelial cells, while CD47 expression levels are very similar (our unpublished results). Thus, although TSP1 action in retinal endothelial cells may be related to its antiangiogenic activity, mainly though CD47/eNOS, the action of TSP1 in choroidal endothelial cells is more anti-inflammatory driven, likely mediated through CD36. However, the specific roles of these interactions appear to be vascular bed specific in the eye and may vary from those noted in endothelial cells from other vascular beds, which deserve further exploration. These data indicate that decreasing TSP1 level locally may enhance inflammation during disease pathogenesis.

Inflammation plays a central role in the pathogenesis of nAMD. Consistent with this notion, here we a significant increase in CCL2/MCP-1 in patients with an incomplete response to anti-VEGF therapy was observed. Also noted was a significant increase in patients responsive to treatment having an AA allele in TSP1 SNP rs2228262. This study correlates with preclinical studies showing that mice lacking TSP1 have increased inflammation and CNV in laser-induced model of nAMD [12, 43]. TSP1 is touted for having an anti-inflammatory and anti-angiogenic role in the eye impacting inflammation and angiogenesis with TSP1 mimetic peptides effectively inhibiting CNV in our preclinical studies [12]. Thus, changes in TSP1 levels may have a significant impact on the outcome of anti-VEGF therapy and plasma levels of the pro-inflammatory mediator CCL/MCP-1 may be a useful indicator of potential responsiveness to treatment.

In summary, the data presented here suggests that TSP1 and BIM SNPs impact the efficacy of anti-VEGF treatment, most likely by their influence on overall inflammation and its clearance. Although location and systemic levels of inflammation are not necessarily the same, here we observed increased plasma CCL2/MCP-1 levels in patients with an incomplete response coupled with responsive patients having the BIM CT allele and TSP1 rs1478604 TT allele. This suggests that mononuclear phagocyte recruitment and clearance capabilities of individual patients may need to be considered for treatment regimens to be most effective. Our studies also suggest the use of CCL2 inhibitors or receptor antagonists (CCR2 antagonists) could be efficacious, either alone or in combination with anti-VEGF for treatment of nAMD patients with high levels of CCL2 and non-responsive to anti-VEGF monotherapy. Thus, further study in this area may improve patient outcomes, preventing vision loss.
